# A Classical Presentation of Optic Disc Pits With Complex Maculopathy

**DOI:** 10.7759/cureus.32469

**Published:** 2022-12-13

**Authors:** Sri Lekha Rao, Archana R Thool

**Affiliations:** 1 Ophthalmology, Jawaharlal Nehru Medical College, Datta Meghe Institute of Medical Sciences, Wardha, IND

**Keywords:** csf, oct, macular edema, congenital anamoly, maculopathy, optic disc pit

## Abstract

A rare congenital abnormality of the optic disc, known as the optic disc pit (ODP), can cause progressive vision loss when it is associated with maculopathy. Only 15% of ODP cases are bilateral, with a reported incidence of 1 in 11,000 cases and with no gender differentiation. A 56-year-old woman presented in the outpatient department with a history of gradual painless diminution of vision in the right eye for one year. On ocular examination of the right eye, the patient has a vision of counting finger 1 m not improving with pinhole, and anterior segment evaluation is within normal limits. On fundus examination, we could appreciate a grayish, white small hypopigmented depression present in the inferotemporal part of the optic disc suggestive of ODP and edema present in the inferior half of the macula along with cystoid macular edema. The fundus photo of the right eye was suggestive of inferotemporal ODP in the right eye and normal fundus in the left eye. Optical coherence tomography (OCT) of the right eye showed inferotemporal ODP connecting with the subarachnoid space of the optic nerve. The macula showed diffuse edema extending from disc to macula, neurosensory detachment, macular schisis, and cystoid macular edema. We report a case of unilateral ODP maculopathy (ODP-M), which is a congenital anomaly of the optic nerve head (ONH) located at the inferotemporal part of the optic disc with multilayered separation involving all retinal layers and connecting with the subarachnoid space, as seen on OCT.

## Introduction

A rare congenital abnormality of the optic disc, known as the optic disc pit (ODP) [1], can cause progressive vision loss when it is associated with maculopathy. The failure of the fetal fissure to close during development is assumed to be the cause of ODPs [2]. Only 15% of ODP cases are bilateral, with a reported incidence of 1 in 11,000 cases and with no gender differentiation [2-4]. Unilateral ODP is inherited in an autosomal dominant fashion [5]. The ODP is clinically present from birth to the ninth decade of life but typically manifests in the third and fourth decades of life because, at this age, progressive vitreous detachment initially appears. Vitreous traction causes the development of ODP-maculopathy (ODP-M). Following the complete posterior vitreous detachment (PVD), there can be a spontaneous resolution of ODP-M. The majority of instances are sporadic, and there are no clear variables that have been linked to the onset of maculopathy. The most common outcome of ODPs is serous detachments at the macula, retinal pigment epithelium (RPE) mottling, and general cystic changes.

The patient does not manifest any symptoms until the onset of ODP-M, which is developed due to fluid accumulation in various retinal layers that may or may not be accompanied by neurosensory separation. Serous macular detachment (SMD) and retinal schisis are characteristic features of ODP-M [6-8]. The movement of fluid from the vitreous into the subretinal space is assumed to be a result of pressure differences within the eye [7,8]. A pressure gradient may exist in an eye with ODP because intracranial pressure is transmitted through cerebrospinal fluid (CSF). As a result, when cerebral pressure is low, vitreous fluid is drawn into the ODP, and when it is high, the fluid is pushed back into the eye, and this will dissect beneath or inside the retina.

Lincoff et al. described the process of retinal fluid accumulation and the sequence of its formation regardless of the source of fluid or the precise pathophysiological mechanism causing ODP-M [7]. First, the fluid present in the ODP causes an inner retinal separation that resembles a schisis, causing mild cecocentral scotoma, followed by an outer layer macular hole behind the inner layer leading to central scotoma. Lastly, there is outer retinal detachment due to subretinal fluid dissection. Various studies regarding the source of fluid and macular alterations have been put forth, although the precise formation of ODPs leading to macular detachment is unknown [9]. The vitreous humor, CSF from the subarachnoid space, leaky blood vessels at the base of the ODP, peripapillary atrophy, and the choroid through the Bruch's membrane are four potential sources of subretinal fluid [10].

In ODP-M, intraretinal and subretinal fluid will accumulate at the macula. Despite improvements in fundus imaging, the fluid's origin is still uncertain, and the exact pathogenesis of the maculopathy is not fully understood.

## Case presentation

A 56-year-old woman presented in the outpatient department with a history of gradual, painless diminution of vision in the right eye for one year. No other ocular complaints were present in both eyes. The patient had no history of systemic illness. The patient did not give any significant family history.

On ocular examination, the patient's right eye had a vision of finger counting 1 m, not improving with pinhole. The anterior segment evaluation was within normal limits. The pupil was reacting to direct and consensual light reflexes. A cortical cataract was seen in the right eye. The left eye had 6/6 vision, and the anterior segment evaluation was within normal limits. The pupil was reacting to direct and consensual light reflexes. A fundus examination was done on slit-lamp biomicroscopy along with a 78 D lens and indirect ophthalmoscopy along with a 20 D lens. In the right eye, a grayish, white small hypopigmented depression was present in the inferotemporal part of the optic disc, suggestive of an ODP. No abnormality in blood vessels was seen. The peripapillary area was normal. There was evidence of edema present in the inferior half of the macula along with cystoid macular edema. The peripheral fundus was normal. In the left eye, the optic disc, blood vessels, macula, and periphery were normal.

**Figure 1:**
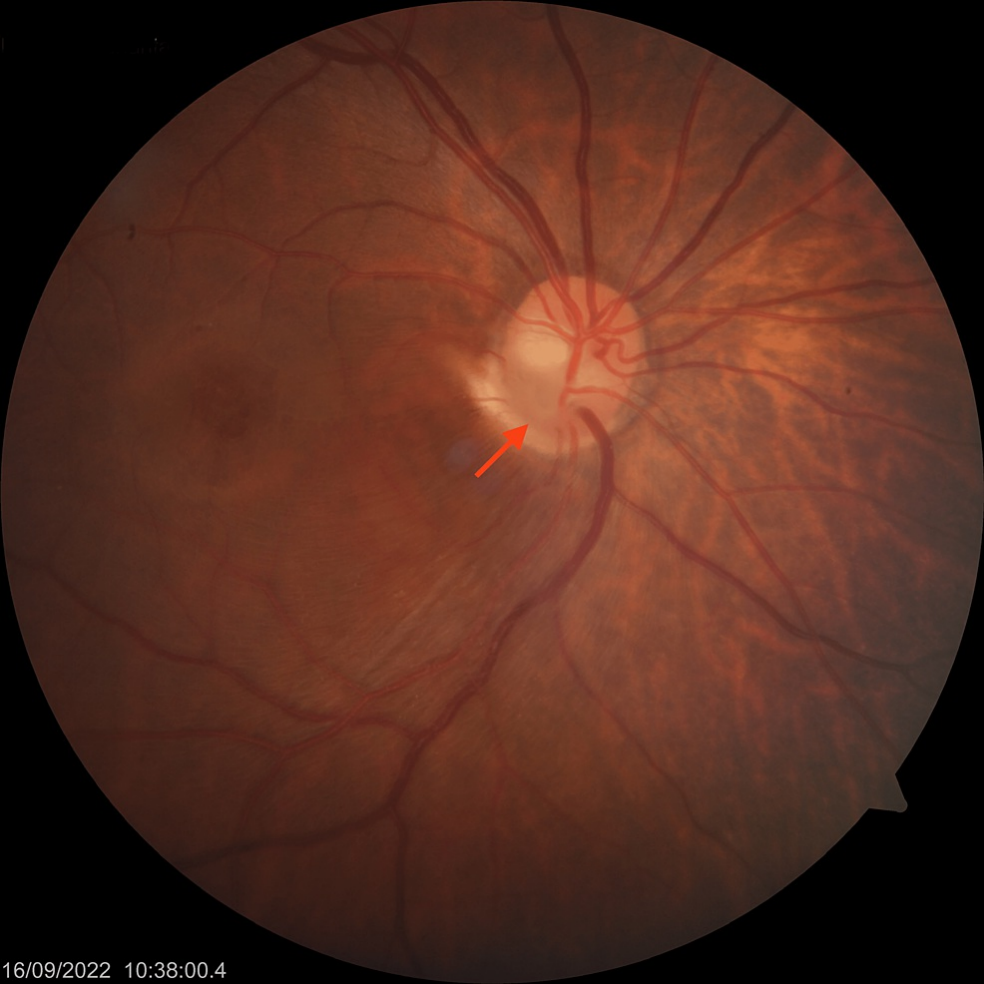
Right eye fundus photo showing inferotemporal optic disc pit.

**Figure 2:**
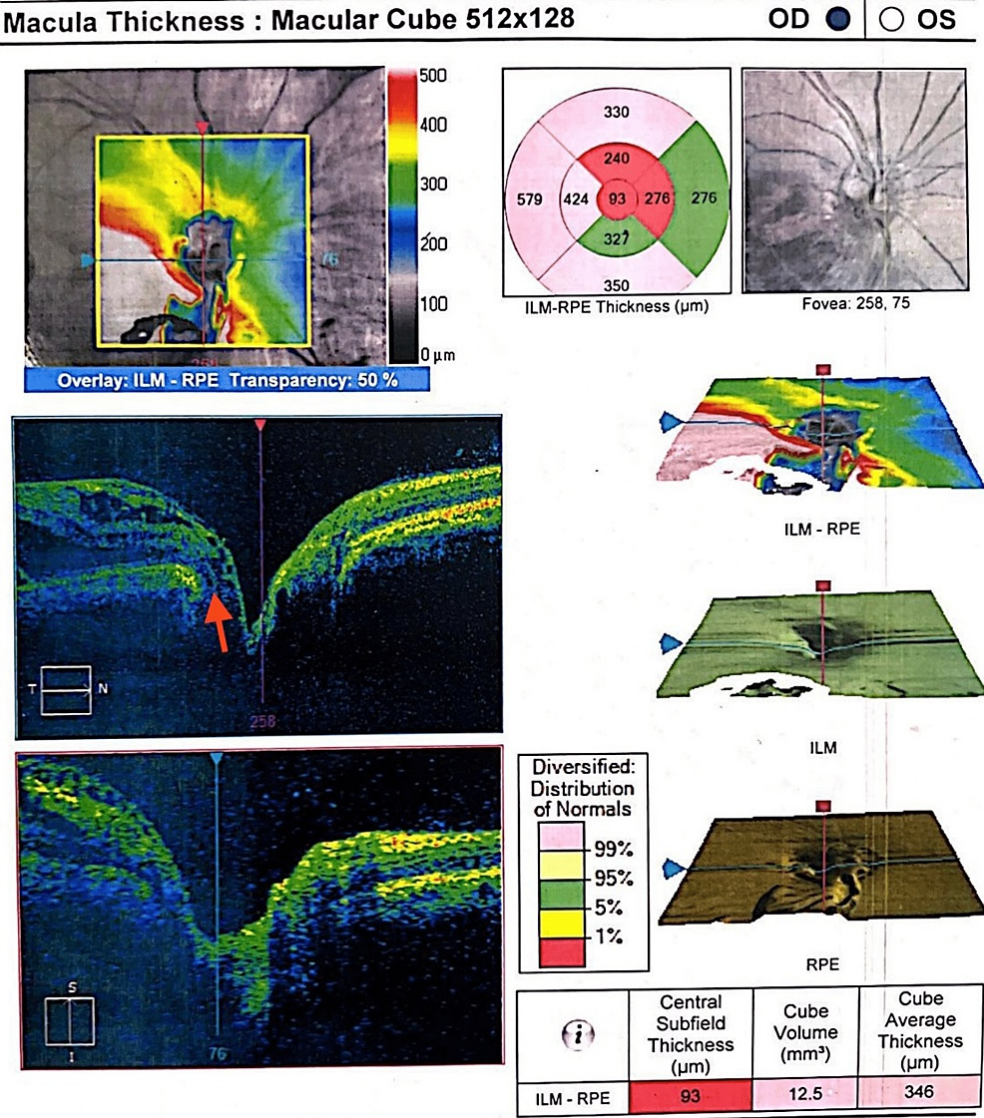
Right eye OCT showing the optic disc pit involving the subarachnoid space of the optic nerve. OCT, optical coherence tomography

**Figure 3:**
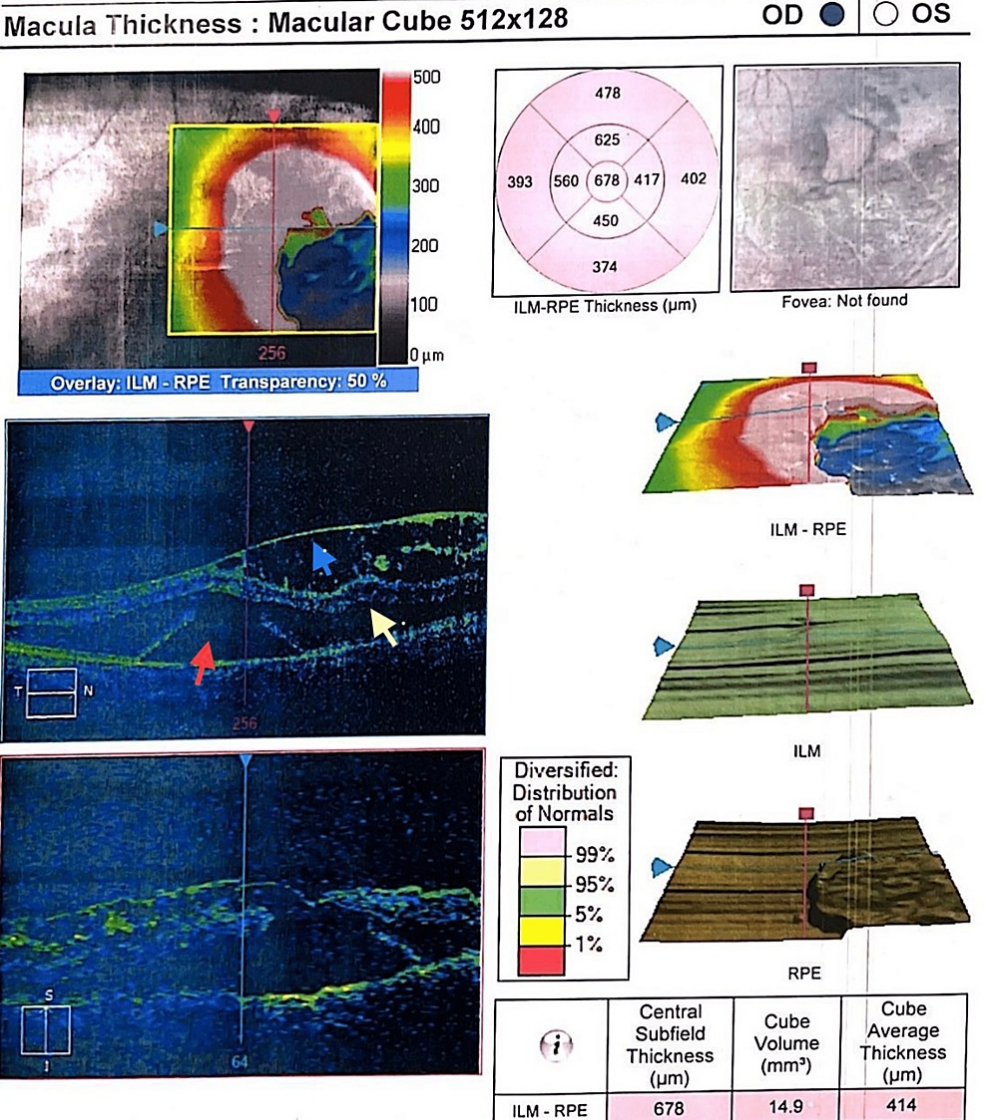
Right eye OCT showing diffuse edema extending from disc to macula, red arrow showing neurosensory detachment, yellow arrow showing macular schisis, and blue arrow showing cystoid macular edema. OCT, optical coherence tomography

The fundus photo of the right eye (Figure 1) was suggestive of an inferotemporal ODP in the right eye. OCT optic disc and macula were done to confirm the diagnosis and involvement of the macula. Right eye OCT (Figure 2) showed an inferotemporal ODP connecting with the subarachnoid space of the optic nerve. In the right eye, the OCT macula (Figure 3) showed diffuse edema extending from the disc to macula, neurosensory detachment, macular schisis, and cystoid macular edema. Left eye OCT appears to be normal.

## Discussion

An ODP is a rare congenital optic disc anomaly that is frequently referred to as a primary, open-angle glaucoma suspect. Lamellar macular holes, full-thickness macular holes, and RPE atrophy, which are typically accompanied by a low visual acuity of 20/200 or worse, are additional retinal findings in long-standing ODP-M [8,11]. It is commonly seen in women than in men. Some patients with optic pits remain asymptomatic throughout life, but 25% to 75% present with ODP-M by 30 to 40 years of age. About 48% of patients can have ODPs in both eyes, which can be associated with scotomas. The prevalence of acquired ODP is less in patients with high-tension glaucoma when compared with low-tension glaucoma. Disc hemorrhages are commonly seen in glaucoma patients with acquired ODP than in others. Commonly, an ODP is seen in the inferotemporal region with or without involving the macula. We report a classical case of an ODP present in the inferotemporal region, which is a rare but usual anatomical site. When left untreated, ODP‐M leads to cystic degeneration [2] of the retina with pigmentary changes and poor visual recovery. There are theories supporting the migration of both vitreous (liquefied form) and CSF through the link between the ODP with the subarachnoid space [12], but the pathophysiology of ODP-M is still poorly understood [13-14]. A study by Arora et al. presented an ODP case with multilayered separation of retinal layers along with neurosensory detachment between the optic disc and fovea, which is similar to our case report. The main goal in the treatment of ODP-M is to promote fluid resorption and avoid fluid migration into the macula. These include pars plana vitrectomy, along with gas tamponade, internal limiting membrane peel, inner retinal fenestrations, PVD, laser photocoagulation to the inferotemporal margins of the optic disc, and mechanically plugging the ODP. The patient was advised pars plana vitrectomy with a fluid tap as a treatment plan and for diagnostic purposes by CSF analysis. The patient was denied surgery and was lost for follow-up.

## Conclusions

We report a case of unilateral ODP, which is a rare congenital anomaly of the optic nerve head (ONH). In our case, the ODP is located in the inferotemporal part of the optic disc, which is a classical site. The pit is seen to be connected with the subarachnoid space on OCT. Also, this patient had ODP-M, causing poor visual acuity. The source of retinal fluid might be CSF in this case, as seen in OCT, but needs analysis by fluid tap to confirm.
